# Design and preliminary application of affinity peptide based on the structure of the porcine circovirus type II Capsid (PCV2 Cap)

**DOI:** 10.7717/peerj.8132

**Published:** 2019-12-05

**Authors:** Junfang Hao, Fangyu Wang, Guangxu Xing, Yunchao Liu, Ruiguang Deng, Hao Zhang, Anchun Cheng, Gaiping Zhang

**Affiliations:** 1 Research Center of Avian Diseases, College of Veterinary Medicine, Sichuan Agricultural University, Chengdu, China; 2 Henan Key Laboratory for Animal Immunology, Henan Academy of Agricultural Sciences, Zhengzhou, China; 3 College of Life Science, Henan Agricultural University, Zhengzhou, China

**Keywords:** Affinity peptides, PCV2 Cap, Molecular docking virtual screening, Purification

## Abstract

**Background:**

Affinity peptides, as a core part of affinity chromatography, play an important role in the purification of target molecules.

**Methods:**

Here we describe the use of molecular docking technology for virtual screening of affinity peptides that specifically recognize the PCV2 Cap protein for the first time. Thirteen candidate peptides with high scores were obtained and then further characterized. Experimentally, the affinity and sensitivity of the peptides studied were identified by ELISA and LSPR, respectively. In order to investigate the purification effect of a selected peptide (L11) for the recombinant PCV2 Cap protein, it was coupled to NHS agarose magnetic beads as an affinity adsorbent (NaMB-L11); and the ligand density of the affinity adsorbent and pH value in the purification of the recombinant PCV2 Cap protein were optimized.

**Results:**

Our data showed that the peptide L11- DYWWQSWE has the smallest K_D_ = 103 nM with higher specificity for PCV2 Cap protein recognition. The NaMB-L11 affinity adsorbent yielded a purified Cap sample with 98% purity at 90% recovery in a single step.

**Conclusion:**

Based on the structure, we obtained a high affinity peptide L11 binding to the PCV2 Cap protein by molecular docking technology. It not only provides a theoretical basis for the design of PCV2 Cap affinity peptide, but a new method for the purification of the PCV2 Cap protein.

## Introduction

As a new type of affinity bionic ligand, affinity peptides can break through the limitations of some traditional ligands and become a research hotspot in recent years (Wang et al., 2016a). Commonly used affinity ligands are metal chelates (Serpa et al., 2005), antigens (Wang et al., 2005), nucleic acids (Cheng et al., 2016) and peptide ligands (Kuo, Lin & Chen, 2016). Among them, peptide ligands have many characteristics such as lower molecular weight, ease of synthesis, good safety profile, higher surface density, and more amenable to be engineered at the molecular level (Lowe et al., 1992). In view of a series of excellent properties of peptide ligands, affinity adsorbents with peptide ligands have been developed as the main technology for separation and purification of target proteins (Fang, Lin & Yao, 2018).

To obtain the top performing affinity peptides rapidly, many screening methods have been developed such as combinatorial chemistry (Liu, Li & Lam, 2017), mRNA display (Roberts & Szostak, 1997), phage display (Smith, 1985), and computer-based virtual screening technology. Most of them are highly dependent on high-throughput experimental screening, which results in heavy workload (Fang, Lin & Yao, 2018; Thirumal Kumar et al., 2017). However, the structure-based molecular docking technology can solve this problem. Molecular docking is one of the important methods of computational virtual screening, which attempts to predict the binding mode and affinity of a ligand to the active site of a protein (Yuriev & Ramsl, 2013). It makes operation simple and fast, reduces the intensity of peptide screening, and shortens the development cycle. Moreover it also greatly improves the screening success rate (Tinberg et al., 2013; Sammond et al., 2011).

PCV2, classified in the family *Circoviridae* and non-enveloped single-stranded DNA viruses, is widespread and has caused huge economic loss for the swine industry around the world every year (Liu et al., 2016; Segalés, 2015). The PCV2 Cap protein is composed of 233∼234 amino acids with an apparent molecular weight of about 26 kDa. It is a unique structural protein and immune protein of PCV2, which is related to its pathogenicity (Hu et al., 2016). Currently, a high dose of PCV2 Cap with high purity is critical for the preparation of good vaccines. However, the common PCV2 Cap purification method, that includes ion-exchange chromatography (Zaveckas et al., 2015), wastes time and energy. Therefore, designing and identifying a peptide ligand with high affinity can provide a potential option for the convenient and efficient purification of PCV2 Cap.

## Materials and Methods

### Materials

Thirteen peptides, named L1∼L13, were synthesized and purified to at least 90% by GL Biochem. (Shanghai, China). The PCV2 Cap protein, porcine epidemic diarrhea virus S protein (PEDV-S), porcine reproductive and respiratory syndrome virus GP5 protein (PRRSV-GP5) and the polyclonal antibodies of PCV2 Cap (anti-Cap) were generated in our laboratory. A carboxyl sensor chip was purchased from Nicoya (Beijing, China). The streptavidin conjugated to horseradish peroxidase (SA-HRP) was purchased from Bioss Biological Engineering (Beijing, China). The goat anti-mouse IgG conjugated to horseradish peroxidase (IgG-HRP) was purchased from Abbkine (Wuhan, China). The NHS-activated magnetic agarose beads (NaMB) were purchased from Enriching Biotechnology (Shanghai, China). Bovine serum albumin (BSA) was purchased from Saibao Biotechnology (Yancheng, China).

### Molecular docking virtual screening

(1) Preparation of protein crystal structure. The 3D structure of PCV2 Cap (PDB ID: 3R0R) (Khayat et al., 2011) was obtained from the protein data bank. First, the PCV2 Cap protein was analyzed by the option of Prepare protein structure of the SYBYL software (Tripos, USA). Backbone, side-chain, termini treatment, and protonation type were fixed following its suggesting. Hydrogens and charges were added. Second, in the stage minimization option, Biopolymer Hydrogens, Waters, and Side-chains were minimized 100 times per step. Third, in the Surflex-Dock-Define SFXC File option, the Threshold value was 0.5, the Bloat value was 10, and the remaining parameters were default values. Finally, the Surflex-Dock Screen (SFXC) File was generated for next docking.

(2) Virtual peptide library design. The initial conformation of the docking peptide was constructed using the Biopolymer/Build/Build Protein module in the SYBYL-X2.0 software. All peptides were designed as a series of linear peptides with different lengths. The peptide library was written to a single file with a library capacity of 16,000. Then, the hydrogen atoms were added and MMFF94 charges were loaded. The peptide was minimized by 1,000 cycles using the MMFF94 force region to obtain its optimum conformation (Liu et al., 2007).

(3) Molecular docking. Molecular docking simulation was carried out with the program FlexX Docking Suite embedded in the SYBYL package. PCV2 Cap was prepared by Preparing Protein Structure embedded in the SYBYL (Rarey et al., 1996).

(4) Evaluation of results. To assess the binding affinity in the docking of the ligand and protein, the Consensus Score (CScore) was referred to as the most criterion (Yu et al., 2018). CScore (Welch, Ruppert & Jain, 1996) is a consensus score function for ligand–receptor affinity. It integrates four scoring functions that include D Score (Jones et al., 1997), C Score (Eldridge et al., 1997), G Score (Kuntz et al., 1982), and PMF Score (Muegge & Martin, 1999) to somehow reduce the bias caused by scoring. Based on the criterion of virtual screening (CScore ≥ 5), we obtained 67 peptide sequences. In SYBYL, CScore always reports the output of the docking engine as total scores.

### ELISA assay for affinity and specificity

The ELISA plate was coated with 50 µL of a 10 µg/mL purified Cap protein solution and incubated for 12 h at 4 °C. Blocking buffer containing 1% BSA was incubated for 1 h at 37 °C. After washing with PBST (PBS containing 0.5% Tween20), biotin (bio)-labeled peptides (1 µg/mL) were separately added and incubated for 30 min at 37 °C. The plate was washed as before, SA-HRP (1:1,000) and IgG-HRP (1:5,000) were separately added and incubated for 30 min at 37 °C. Meanwhile, Phosphate Buffer Saline (PBS) and anti-Cap antibodies were used as negative and positive controls, respectively. The reaction was developed by using 3,3,5,5-tetramethylbenzidine (TMB) as substrate. The OD value of each well was measured at 450 nm using an ELISA plate reader.

The P/N value and cross-reactivity of the candidate peptide were determined according to the ELISA method described above. When the ratio between P/N [(OD_sample_-OD_blank_)/(OD_negative_-OD_blank_)] is more than 2.1, the peptides exhibiting high specificity were chosen for posterior experiments using pET28a as negative control. Meanwhile, the cross-reactivity of affinity peptides was evaluated using proteins outside the circovirus genus, namely PEDV-S protein, PRRSV-GP5 protein, and BSA. Their coating concentration was 10 µg/mL.

### Kinetic dissociation measurements

The equilibrium binding constant (K_D_) of peptides was determined using an Open SPR instrument (Nicoya, Canada). First, purified Cap protein was covalently immobilized on a carboxyl Au colloidal nanoparticles chip. Second, the running buffer (PBS, pH 7.4) was flowing through the chip (for preparation of the PCV2 Cap protein biochip; see ESI 1) until a smooth testing baseline was obtained. Then, all peptides were respectively dissolved in PBS at predetermined concentrations and injected to detect the resonance signal changes. In each cycle, 300 µL of peptide solution was injected into the flow cell of the Cap chip for 5 min. Finally, the data were assessed to determine the association constant (k_a_, 1/Ms), the dissociation constant (k_d_, 1/s), the equilibrium binding constant (K_D_, M), and K_D_ = kd/ka via Trace Drawer 1.7 (Nicoya, Canada).

### Effect of pH

First, the NaMB-L11 affinity adsorbent was prepared (see ESI 2). Next, the NaMB-L11 (100 µL) was added into 100 µL of recombinant PCV2 Cap solution (for the expression of the recombinant PCV2 Cap protein; see ESI 3) with different protein concentrations (0.5∼6 mg/mL) at various pH values (10, 9, 8, 7, 6, 5, and 4). The mixtures were then shaken 2 h at 25 °C. The amount of adsorbed protein was calculated according to a mass balance Langmuir equation was fitted to the adsorption isotherm. The formula is as follows:

\begin{eqnarray*}{\mathrm{Q}}^{\mathrm{\ast }}= \frac{{\mathrm{Q}}_{\mathrm{m}}\times {\mathrm{c}}^{\mathrm{\ast }}}{{\mathrm{K}}_{\mathrm{d}}+{\mathrm{c}}^{\mathrm{\ast }}} \end{eqnarray*}

Where Q* and C* are the equilibrium adsorption capacity of resin (mg/g resin) and the equilibrium protein concentration in the liquid phase (mg/mL), respectively. Q_m_ represents the saturated adsorption capacity (mg/g resin), while Kd is the apparent dissociation constant (mg/mL).

### Effect of ligand density

First, the affinity adsorbent NaMB-L11 with different ligand densities was prepared (see ESI 2). Second, the effect of different ligand densities on the adsorption of PCV2 Cap protein was measured by the same method as above. Third, the NaMB-L11 (100 µL) with different ligand densities (see ESI 4) was added to 100µL of recombinant PCV2 Cap solution (PBS, pH 9) containing different concentrations (0.5∼6 mg/mL). Subsequently, the adsorption operation until reaching equilibrium was carried out as described above. Finally, SDS–PAGE was used to monitor the purification effect. The purity of PCV2 Cap protein was analyzed by gel-pro Analyzer. The recovery rate was calculated using a micro-BCA Kit (Thermo Fisher Scientific).

### Analysis of PCV2 Cap protein immunoactivity after elution

Frist, the PCV2 Cap protein sample was eluted using 100 mM glycine-hydrochloric acid (pH 2.4), and then the sample was neutralized with 100 mM Tris–HCl (pH 8). Second, protein samples were separated by 12% SDS-PAGE. Third, the protein samples were transferred to a polyvinylidene fluoride (PVDF) membrane that was blocked with 5% non-fat milk for 1 h at 37 °C. Then, the anti-Cap was added and incubated for 12 h at 4 °C. Subsequently, the PVDF membrane was washed 5 times with PBST and incubated with a secondary antibody for 1 h at room temperature. Finally, the detection was performed using an Enhanced Chemiluminescent (ECL) kit (NCM, Suzhou, China).

### Statistical analysis

All analyses were conducted using GraphPad Prism version 5.0 software and Excel. Values were expressed as mean ± SEM. Cross-reactivity between groups was performed using a two-way ANOVA. Statistical significance was determined at *P* ≤ 0.05 (*), *P* ≤ 0.01 (**), or *P* ≤ 0.001 (***).

## Results

### Molecular docking virtual screening

**Figure 1:**
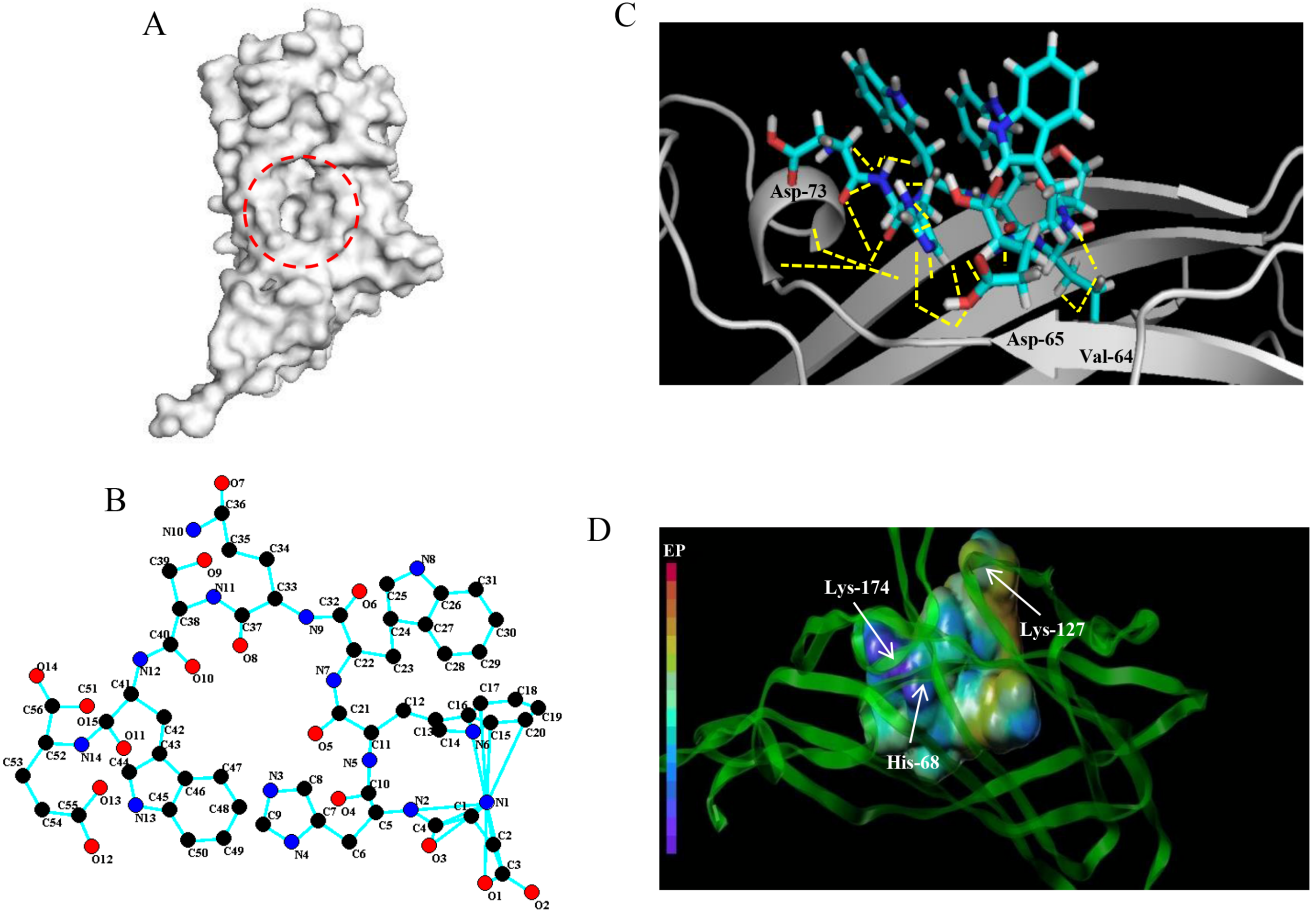
Docking of affinity peptide L11 on the PCV2 Cap protein. (A) The active pocket of PCV2 Cap protein (PDB:3R0R), as in red dotted frame. (B) The 2D structure of L11-DYWWQSWE affinity peptide. (C) The key residues and hydrogen bonds (yellow dotted line) of the interaction between the L11 peptide with the PCV2 Cap protein. The graphics were developed by Pymol –0_99rc–bin-win32. (D) The charge interaction between the L11 peptide and the active site of PCV2 Cap protein. The peptide L11 and PCV2 Cap protein are shown in a spherical and ribbon model, respectively. The electrostatic potential ranges from red (most positive ) to purple (most negative).

Based on the detection of active pockets, the amino acid region at positions 63-120 of the PCV2 Cap protein was predicted as docking pockets (Fig. 1A). In this article, the affinity peptide length was randomly assigned to 2∼9 amino acids because peptide ligands are rarely outside these boundaries (Jeziorna et al., 2014). Meanwhile, through preliminary screening of the experiments (see ESI 5), we obtained and synthesized 13 potentially high affinity candidate peptides from 67 peptide sequences.The 13 peptide sequences and CScore values are listed in Table 1. The L11 affinity peptide consists of two uncharged amino acids (Ser and Gln), two acidic amino acids (Asp and Glu), and two hydrophobic amino acids (Tyr and Trp). Its 2D structure is shown in Fig. 1B. The hydrogen bond (yellow dotted line) of the interaction between L11 peptide and PCV2 Cap is shown in Fig. 1C. The key amino acids at the binding site of PCV2 Cap protein are 64-Val, 65-Asp, and 73-Asp. From the analysis of the charge interaction between L11 peptide and the active site of PCV2 Cap protein (Fig. 1D), it was found that Glu of peptide L11 had strong intermolecular electrostatic attraction with His-68 and Lys-174 of PCV2 Cap, respectively, and there was also strong intermolecular electrostatic attraction between Asp and Lys-127 of PCV2 Cap. Therefore, the electrostatic interaction may play an important role in the binding of peptide L11 and PCV2Cap.

**Table 1:** Sequence and CScore values of thirteen candidate peptides obtained by FlexX/SYBYL virtual screening.

Peptide No	Amino acid sequence	CScore
L1	FREWKK	11.16
L2	KHKFWQR	13. 53
L3	KKHKFWQS	12.04
L4	WQGMMKEE	13.39
L5	FQSWE	12.16
L6	HWWRDPC	11. 53
L7	RKHKRWK	12.04
L8	RKHEHHE	13.38
L9	WQSWEWTG	13.96
L10	DRWSHE	12.19
L11	DYWWQSWE	14.37
L12	KRWKHQSW	12.32
L13	KWWQSWEW	12.67

### ELISA analysis affinity and specificity

**Figure 2:**
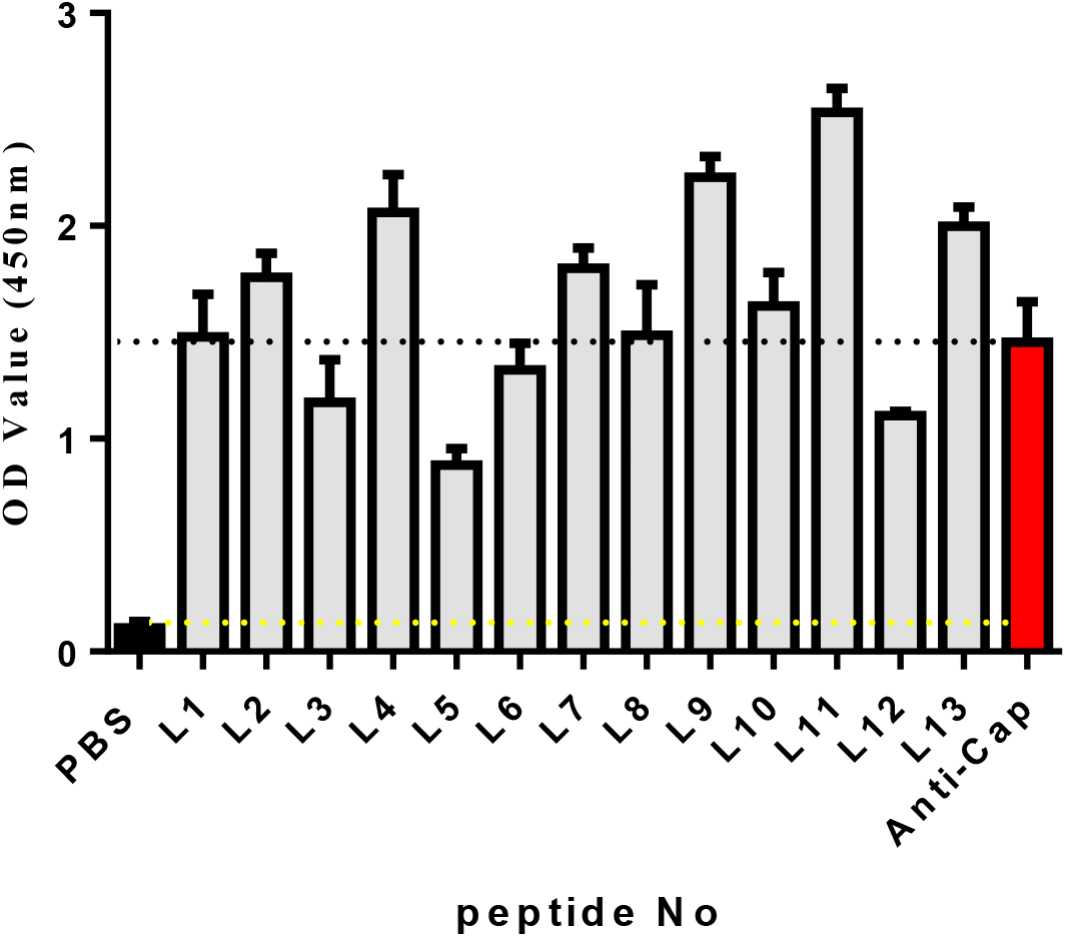
The affinity of each peptide binding with the PCV2 Cap protein was analyzed via ELISA.

Known from the ELISA results (Fig. 2), the OD_450_ values of 13 candidate peptides were obtained ranging from 0.836 to 2.534. Among them L4, L7, L9, L11, and L13 had the best ability to bind with Cap. As a result, the above five peptides were selected for further analysis.

**Figure 3:**
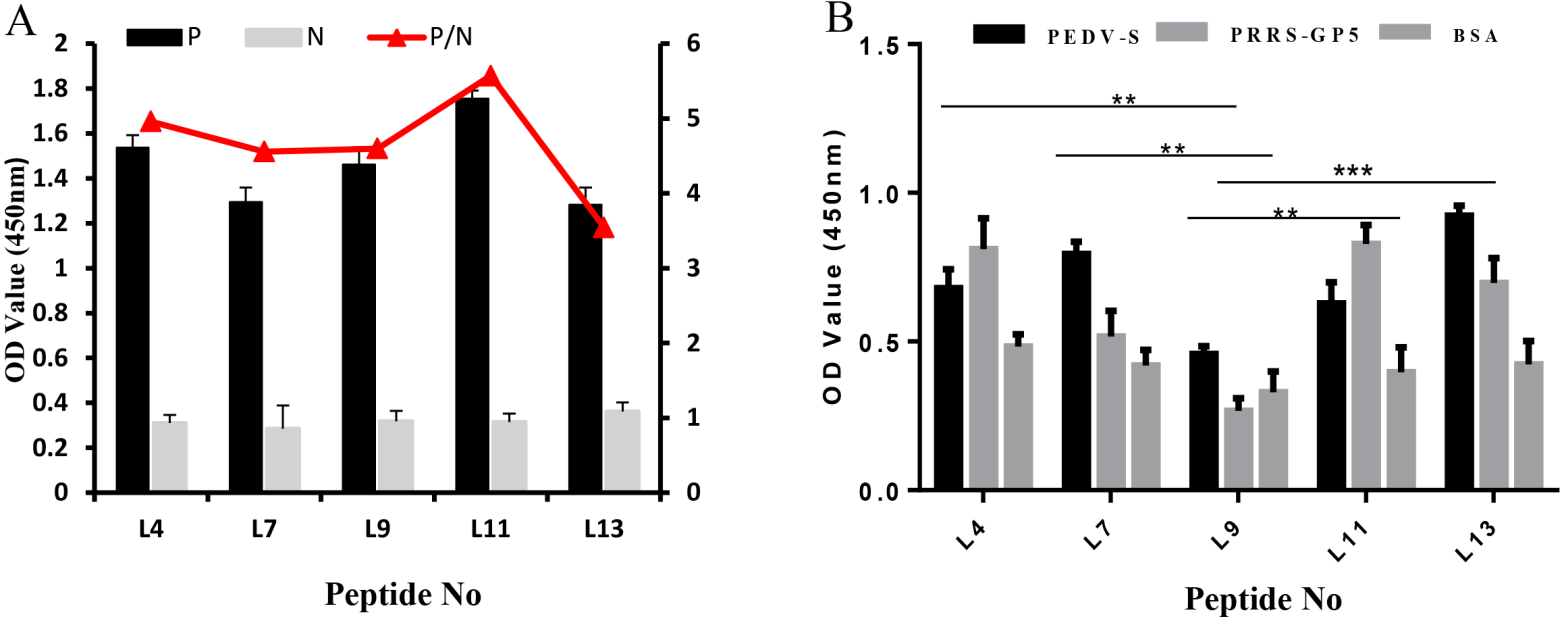
The specificity of each peptide binding with the PCV2 Cap protein was analyzed via ELISA. (A) The P/N values of five peptides. The P/N value represents the binding specificity of the peptides (P: recombinant PCV2 Cap protein; N: pET28a of negative control). (B) The cross-reactivity of five peptides. All the samples were tested through three parallel groups. ^∗∗^*P* ≤ 0.01, ^∗∗∗^*P* ≤ 0.001.

The specificity of these five peptides was evaluated by P/N values and cross-reactivity. When the P/N value is greater than 2.1, the specificity of the peptide is good (Zhang et al., 2018). Figure 3A shows that the five peptides for Cap had P/N values greater than 2.1. Importantly, peptides L4 and L11 are the most promising due to their higher P/N values, 4.96 and 5.57; respectively. For the cross-reactivity of three proteins (Fig. 3B), L9 was significantly different from L4, L7, and L11 peptides (*P* ≤ 0.01), and was also significantly different from L13 (*P* ≤ 0.001). The results indicated that L9 had the lowest cross-reactivity.

### LSPR of affinity peptides analysis

**Figure 4:**
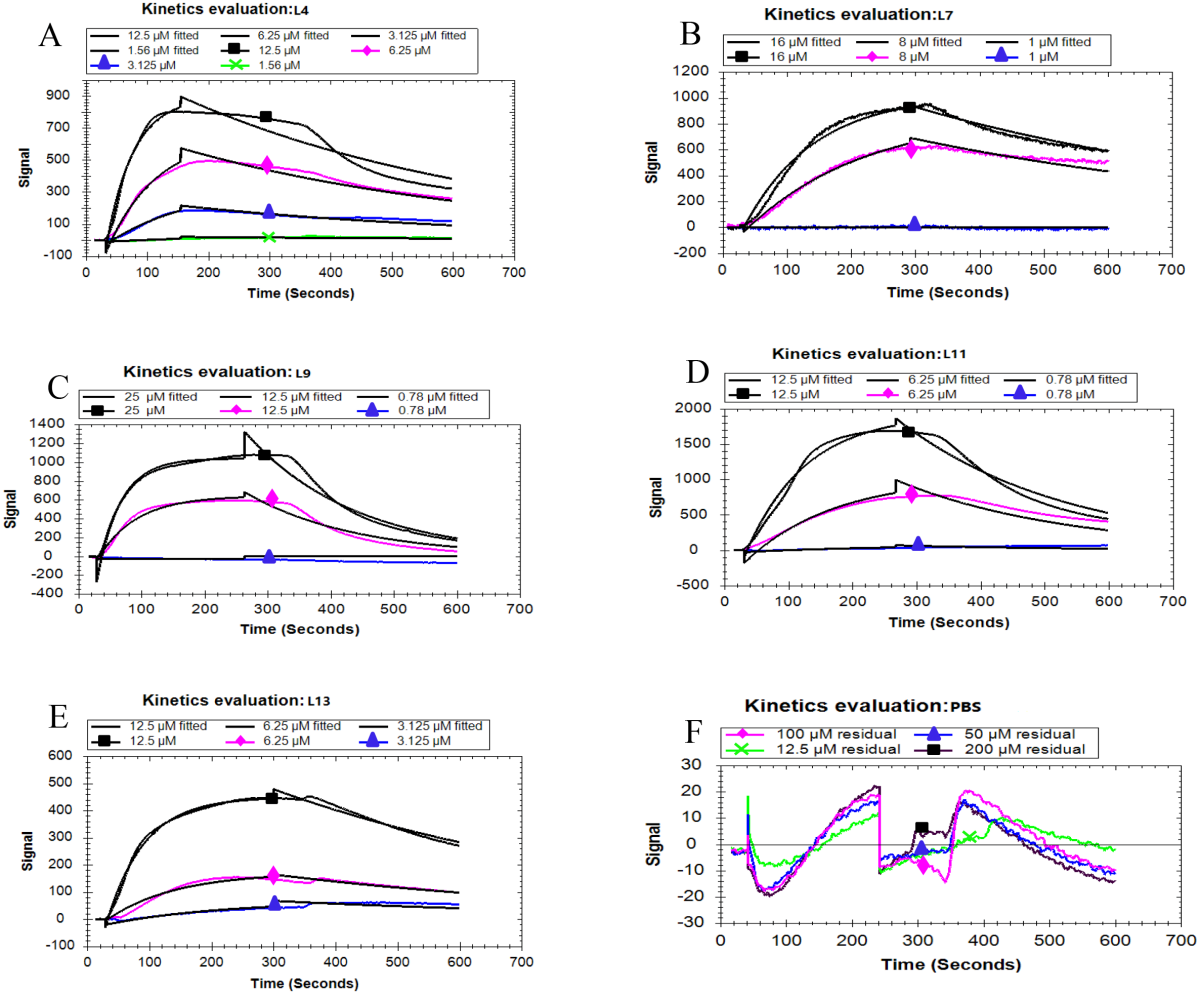
Kinetic analysis between five peptides binding with the PCV2 Cap protein by LSPR. (A–F) represented the association and dissociation curve of L4, L7, L9, L11, L13 and PBS, respectively. Data were fit to a 1:1 interaction model (see ESI. 6) using Trace Drawer 1.7.

Based on the fitted different curves by the 1:1 binding interaction model (Figs. 4A–F), the relevant kinetic data of five peptides associated with the PCV2 Cap, were obtained (Table 2). As can be seen, all the peptides exhibited different binding strength for Cap with various K _D_ values. The L11 peptide had the highest binding affinity compared to other peptides, with a K _D_ of 103 nM. These results demonstrate that the affinity of the five peptides corresponds well with the result in the ELISA. From the mentioned description, we have found that L11 has the highest affinity and specificity, thus it is considered as the best peptide ligand.

### Effect of pH

**Figure 5:**
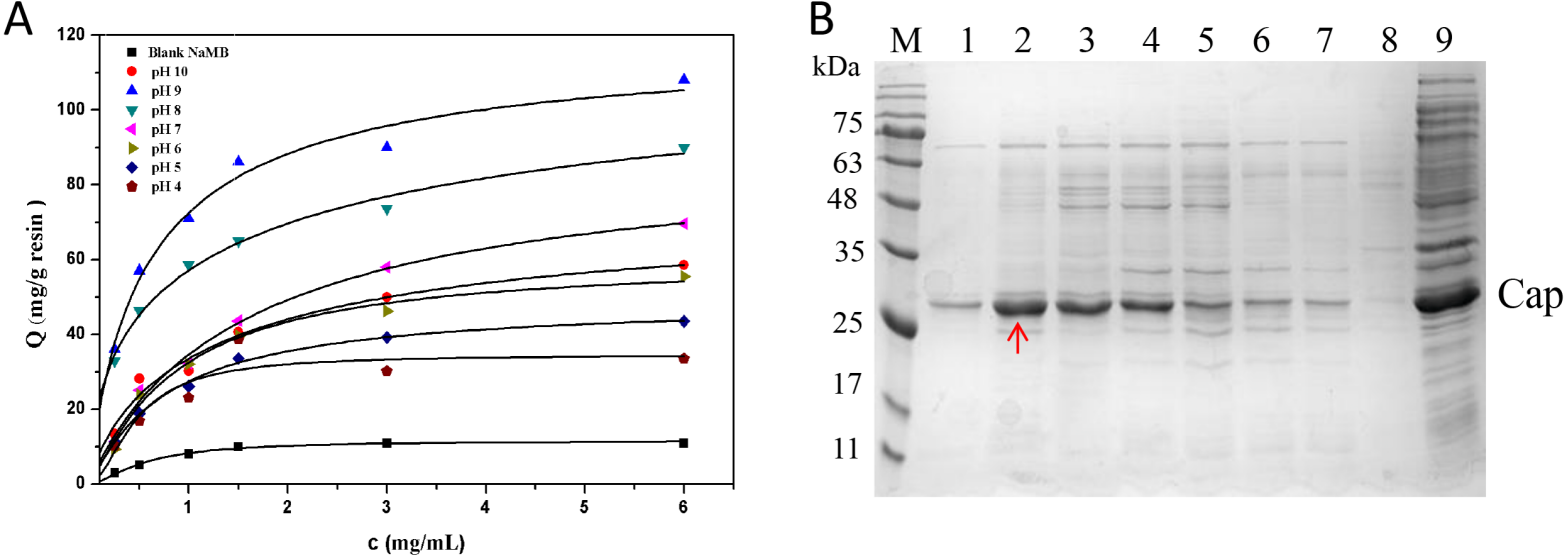
The optimization of pH conditions. (A) Adsorption isotherms at different pH valuess. (B) SDS-PAGE analysis of samples collected at different pH valuess. Lanes 1–7 represent samples collected at pH values of 10, 9, 8, 7, 6, 5, and 4, respectively; Lanes 8–9 represent blank NaMB (BSA-blocked) and recombinant PCV2 Cap protein, respectively.

In Fig. 5A, it is presented that the adsorption capacities of the NaMB-L11 greatly relied on pH. At different pH values, with the increase of sample concentration, the adsorption capacity of the Cap protein increased gradually. The maximum binding capacity could be found at pH 9 (Fig. 5B). The values of Qm and Kd are listed in Table 3. SDS-PAGE analysis that: at pH 9, NaMB-L11 could specifically adsorb Cap protein with the maximum adsorption capacity, while the non-specific binding of contaminants is shown at pH 8-4. The control BSA-blocked NaMB had almost no adsorption of the PCV2 Cap protein. In order to avoid non-specific adsorption, the NaMB need to be blocked with 1% BSA. Because the NaMB will carry some BSA, there is a weak protein band between 63–75 kDa in Fig. 5B corresponding to BSA.

### Effect of ligand density

**Figure 6:**
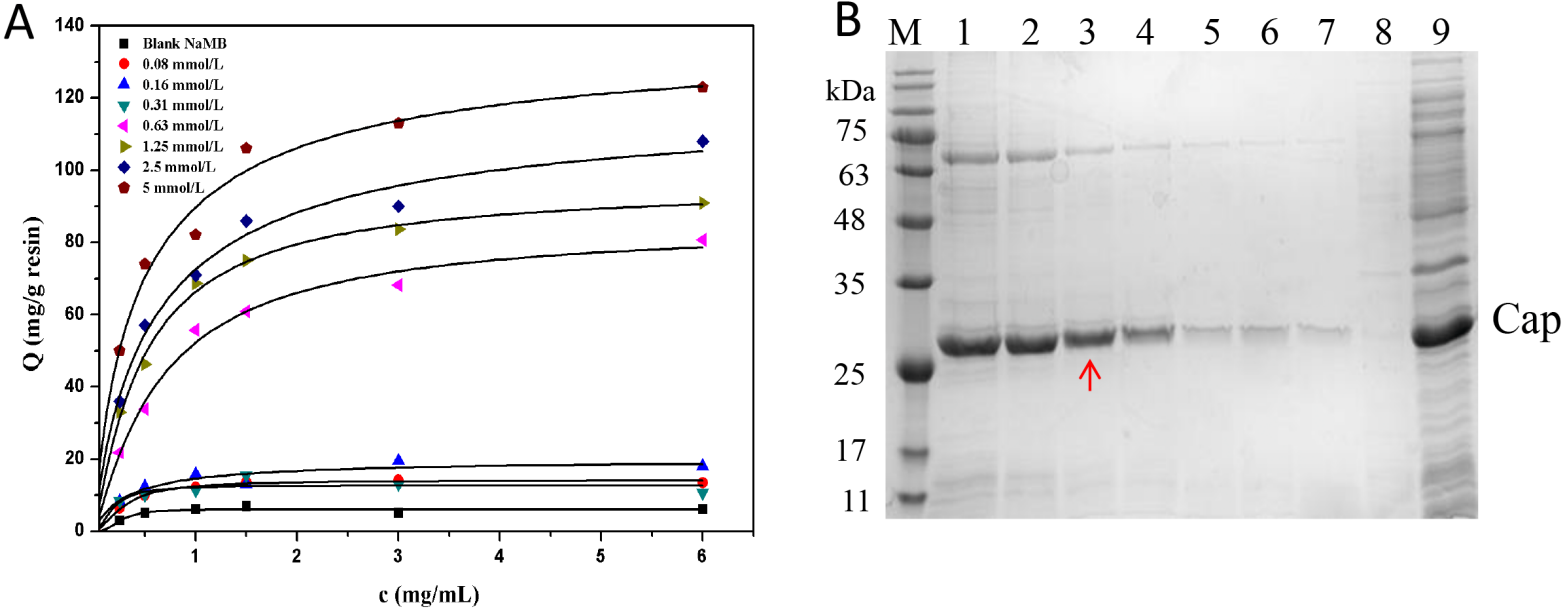
The optimization of ligand density. (A) Adsorption isotherms at different peptide densities. (B) SDS-PAGE analysis of samples collected at different ligand densities. Lanes 1–7 represent samples collected at ligand density of 5, 2.5, 1.25, 0.63, 0.31, 0.16 and 0.8 mmol/L; Lanes 8–9 represent blank NaMB (BSA–blocked ) and recombinant PCV2 Cap protein, respectively.

From Fig. 6A, it is evident that NaMB with higher ligand density at the range tested had higher adsorption capacities. The values of Qm and Kd are listed in Table 4. Figure 6B showed that for the high ligand density region (5∼2.5 mmol/L), increasing the adsorption of Cap protein may also lead to an increase in the adsorption of non-specific substances. For the low ligand density range (0.08∼0.31 mmol/L), the recovery of protein was significantly reduced. For the medium ligand density range (1.25∼0.63 mmol/L), the NaMB-L11 adsorbent presented a better compromise between purity and recovery with a recovery yield of 90% and protein purity of 98%, especially at 1.25 mmol/L. The recovery and purity of PCV2 Cap at each ligand density are shown in Table 5. Thus 1.25 mmol/L was chosen as the best ligand density.

**Table 2:** The kinetic data of affinity peptides-PCV2 Cap interactions in detail. A lower K_*D*_ value indicated a higher affinity of the peptide binding with PCV2 Cap.

Peptide No	k_a_(1/Ms)	k_d_(1/s)	K_D_(M)
L4	1.92 ×10^3^ (±1.09 ×10^2^)	2.36 ×10^−3^ (±5.0 ×10^−5^)	1.23 ×10^−6^ (±8.8 ×10^−8^)
L7	8.56 ×10^2^ (±0.64 ×10^2^)	9.10 ×10^−4^ (±2.3 ×10^−5^)	1.06 ×10^−6^ (±6.72 ×10^−8^)
L9	8.55 ×10^2^ (±0.42 ×10^2^)	5.74 ×10^−3^ (±1.21 ×10^−4^)	6.71 ×10^−6^ (±3.95 ×10^−7^)
L11	1.01 ×10^4^ (±5.61 ×10^2^)	1.04 ×10^−3^ (±2.36 ×10^−5^)	1.03 ×10^−7^ (±8.23 ×10^−9^)
L13	3.35 ×10^2^ (±0.24 ×10^2^)	3.34 ×10^−3^ (±7.28 ×10^−5^)	9.97 ×10^−6^ (±6.45 ×10^−7^)

### Analysis of PCV2 Cap Protein immunoactivity after elution

**Figure 7:**
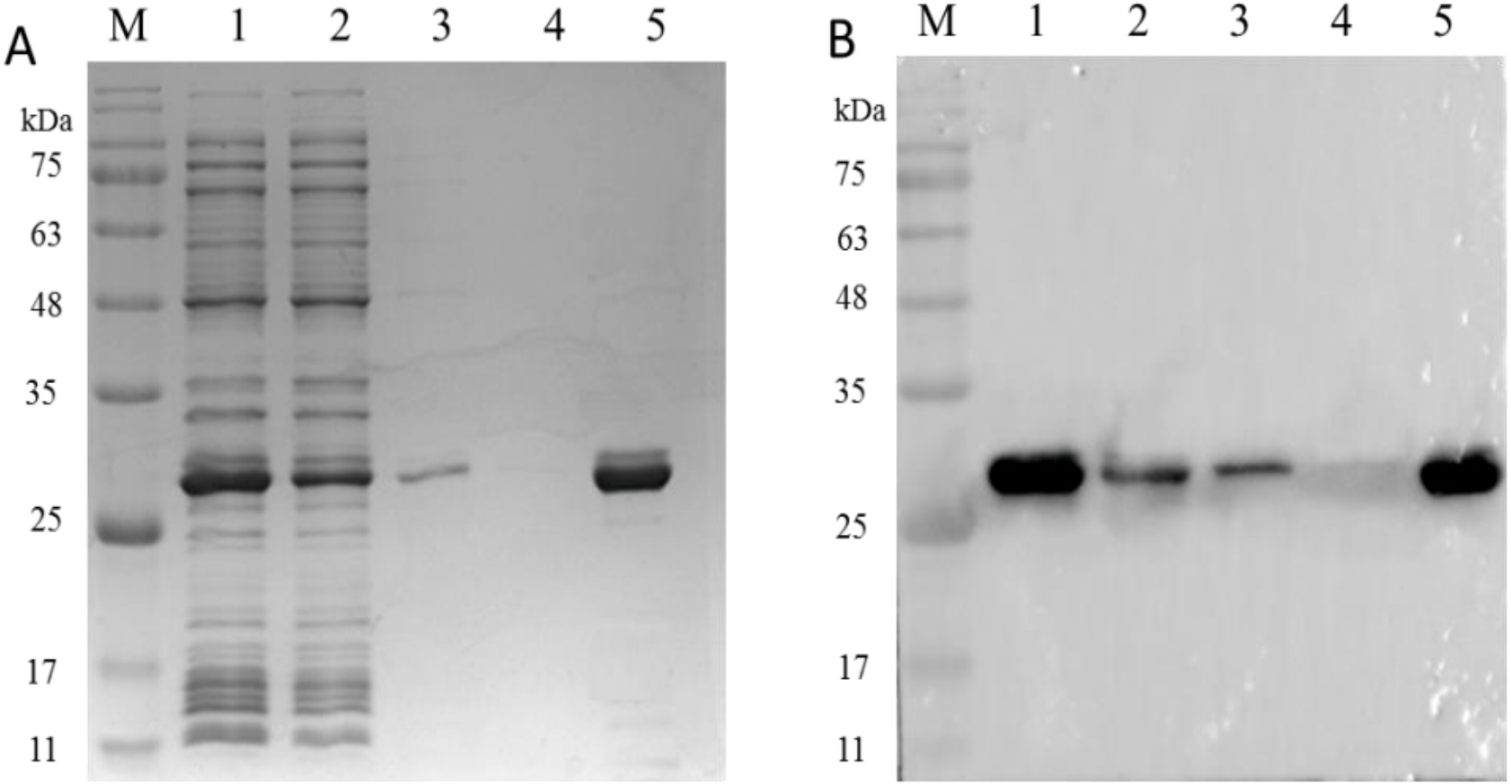
The analysis of PCV2 Cap protein immunoactivity after elution. SDS-PAGE (A) and western blot (B) analysis of the eluted PCV2 Cap protein. Lane 1: the recombinant PCV2 Cap protein before loading; Lane 2: the recombinant PCV2 Cap protein after loading; Lane 3: washing 1; Lane 4: washing 2 and Lane 5: the eluted PCV2 Cap protein.

As shown in Fig. 7A, SDS-PAGE results showed that the PCV2 Cap protein was efficiently dissociated in a glycine-hydrochloric acid solution and a single target band appeared at 26 kDa. As shown in Fig. 7B, WB results showed that the PCV2 Cap protein reacted specifically with anti-PCV2 antibody; indicating that the purified PCV2 Cap had immunogenicity. The results suggested that the NaMB-L11 affinity adsorbents can be used to purify recombinant PCV2 Cap.

**Table 3:** The values of *Q*^*m*^ and K_*d*_ at different pH values.

Sample	Q^m^ (mg/g)	K_d_ (mg/mL)
pH 10	73.2 ± 0.5	3.9 ± 0.4
pH 9	121.6 ± 1.4	1.9 ± 0.3
pH 8	109.0 ± 1.7	2.1 ± 0.2
pH 7	98.5 ± 0.9	2.8 ± 0.5
pH 6	67.1 ± 1.3	4.7 ± 0.3
pH 5	32.0 ± 1.8	6.0 ± 0.8
pH 4	26.0 ± 1.2	6.2 ± 0.6
Blank NaMB	14.0 ± 1.1	8.0 ± 0.5

**Table 4:** The values of *Q*^*m*^ and *K*_*d*_ at different ligand densities.

Sample	Q^m^ (mg/g)	K_d_ (mg/mL)
0.08 mmol/L	39.5 ± 0.9	8.0 ± 0.6
0.16 mmol/L	48.1 ± 0.5	7.2 ± 0.4
0.31 mmol/L	52.6 ± 1.3	6.1 ± 0.2
0.63 mmol/L	70.7 ± 1.1	4.8 ± 0.3
1.25 mmol/L	91.4 ± 0.6	2.4 ± 0.4
2.5 mmol/L	109.0 ± 2.0	2.0 ± 0.3
5 mmol/L	132.0 ± 1.2	1.7 ± 0.2
Blank NaMB	9.0 ± 0.4	8.3 ± 0.5

**Table 5:** The recovery and purity of PCV2 Cap protein at different ligand densities.

NaMB-L11 (Ligand density)	PCV2 Cap purity (%)	Recovery yied (%)
0.08 mmol/L	48 ± 0.6	9 ± 1.5
0.16 mmol/L	61 ± 1.2	17 ± 0.7
0.31 mmol/L	54 ± 2.5	12 ± 1.9
0.63 mmol/L	77 ± 1.8	65 ± 2.3
1.25 mmol/L	98 ± 0.9	90 ± 3.2
2.5 mmol/L	93 ± 1.3	92 ± 2.6
5 mmol/L	86 ± 2.6	95 ± 1.7

## Discussion

With the rapid development of bioinformatics and computational molecular simulation technology, molecular docking technology provides a robust platform for the virtual screening of affinity peptides. In this study, we obtained a peptide ligand L11 with high affinity and specificity for the PCV2 Cap protein by a combination of virtual screening and experimental validation.

CScore is a ligand-receptor affinity score function that integrates several popular scoring functions. While assessing the binding affinities and specificity in the molecular docking of the affinity peptides and target protein, CScore was referred to as the most criterions (Yu et al., 2018). Ouyang, Handoko & Kwoh (2011), confirmed that CScore is able to accurately predict the binding affinity of proteins and ligands for docking. Generally, there are many interactions between the acceptor proteins and the affinity adsorbent, including hydrophobic interactions, electrostatic interactions, and hydrogen bonding (Wang et al., 2014). When analyzing the key residues of the PCV2 Cap protein binding site, we found that only VAL at position 64 plays a hydrophobic role in the interaction between L11 and PCV2 Cap. In addition, the hydrophobic amino acids of the peptide L11 sequence represent only 37%. These results indicated that the hydrophobic interaction is not the main binding force between the peptide ligand and the target protein.

Zheng et al. (2017), using the M13 phage display peptide library technology, obtained the peptide motif of the PCV2 Cap protein after three rounds of biopanning. Nevertheless, its effect on purification of the PCV2 Cap protein has not been reported. Some studies have shown that affinity peptides have a high affinity as part of phage sheath proteins, but when the peptide is synthesized and immobilized; its affinity decreases or even disappears (Gaskin et al., 2001). In this paper, the designed and identified L11 peptide was used as the optimal ligand and conjugated to NaMB as affinity adsorbent.

At present, magnetic particles have been widely used as matrices of affinity adsorbents because of their simple functionalization, strong loading capacity, and convenient magnetic control effect (Yang et al., 2016; Fernandes et al., 2016). The NaMB gel contains NHS groups on its surface that need not be activated before use. The coupling method is simple and efficient; and it is convenient to quickly verify the ability of the affinity peptide to purify the protein. It was found that the adsorption capacity of the NaMB-L11 adsorbent greatly relies on both pH and ligand density. Similar phenomena are also reported in the literature (Lund et al., 2012; Wang et al., 2016b; Zhao, Shi & Sun, 2014). The reasons for the pH dependence in this paper are as follows: at pH 9, which is less than the isoelectric point of PCV2 Cap (pI = 10.84) (Zhao, 2018), protein molecules had a positive charge and the peptide L11 had a negative charge (according to isoelectric.org). Therefore, the electrostatic interaction was the main force for the binding of peptide L11 and PCV2 Cap. Furthermore, ligand density is an important factor relative to the adsorption behavior, which affects the yield and purity of the isolated product to some extent. In other words when the ligand density is increased to a certain extent, the increase in the adsorption of the target substance may be accompanied by a decrease in specificity; resulting in a decrease in purity. In this work when the ligand density of the NaMB-L11 adsorbent was 1.25 mmol/L, the adsorption capacity and purity show a good compromise. Together, pH and ligand density have a great influence on the adsorption of the PCV2 Cap protein and the NaMB-L11 adsorbent can be reasonably prepared and used considering both factors.

In the present research, we have demonstrated, for the first time, the use of a molecular docking technology for virtual screening of affinity peptides that specifically bind to the PCV2 Cap protein. The L11 peptide with high affinity and specificity can be used as the best peptide ligand. Then, the optimal binding conditions (pH and peptide density) of the NaMB-L11 adsorbent for adsorption of the PCV2 Cap protein were establised.

## Conclusion

In this work, we discovered thirteen peptide sequences targeting the PCV2 Cap protein using molecular docking virtual screening technology. The L11 peptide with high affinity and specificity was selected as the optimal ligand, which was coupled to NHS agarose magnetic beads (NaMB-L11) as an affinity adsorbent. In this process, the effect of different pH values and ligand densities was investigated for the purification of the recombinant PCV2 Cap protein. The best affinity adsorbent yielded PCV2 Cap with a 98% purity at 90% recovery. In short, the affinity peptide L11, which can specifically recognize the PCV2 Cap protein, was successfully obtained by molecular docking technology. This technology provides a powerful tool and new insights for the purification of the PCV2 Cap protein in the future.

##  Supplemental Information

10.7717/peerj.8132/supp-1 Supplemental Information 1 Supplemental material

10.7717/peerj.8132/supp-2 Supplemental Information 2 Raw data

## Additional Information and Declarations

### Data Availability

The following information was supplied regarding data availability: The raw data are available in the Supplementary Files.

## References

[ref-1] Cheng D, Yu M, Fu F, Han W, Li G, Xie J, Song Y, Swihart MT, Song E (2016). Dual recognition strategy for specific and sensitive detection of bacteria using aptamer-coated magnetic beads and antibiotic-capped gold nanoclusters. Analytical Chemistry.

[ref-2] Eldridge MD, Murray CW, Auton TR, Paoger GV, Mee RP (1997). Empirical scoring functions: I. The development of a fast empirical scoring function to estimate the binding affinity of ligands in receptor complexes. Journal of Computer-Aided Molecular Design.

[ref-3] Fang Y, Lin D, Yao S (2018). Review on biomimetic affinity chromatography with short peptide ligands and its application to protein purification. Journal of Chromatography A.

[ref-4] Fernandes CS, Barbosa I, Castro R, Pina AS, Coroadinha AS, Barbas A, Roque AC (2016). Retroviral particles are effectively purified on an affinity matrix containing peptides selected by phage-display. Biotechnology Journal.

[ref-5] Gaskin DJ, Starck K, Turner NA, Vulfson EN (2001). Phage display combinatorial libraries of short peptides: ligand selection for protein purification. Enzyme and Microbial Technology.

[ref-6] Hu G, Wang N, Yu W, Wang Z, Zou Y, Zhang Y, Wang A, Deng Z, Yang Y (2016). Generation and immunogenicity of porcine circovirus type 2 chimeric virus-like particles displaying porcine reproductive and respiratory syndrome virus GP5 epitope B. Vaccine.

[ref-7] Jeziorna A, Kazmierski S, Paluch P, Skorupska E, Potrzebowski MJ (2014). Recent progress in the solid-state NMR studies of short peptides: techniques, structure and dynamics. Annual Reports on NMR Spectroscopy.

[ref-8] Jones G, Willett P, Glen RC, Leach AR, Talor R (1997). Development and validation of a genetic algorithm for flexible docking. Journal of Molecular Biology.

[ref-9] Khayat R, Brunn N, Speir JA, Hardham JM, Ankenbauer RG, Schneemann A, Johnson JE (2011). The 2.3-Angstrom structure of porcine circovirus 2. Journal of Virology.

[ref-10] Kuntz ID, Blaney JM, Oatley SJ, Langridge R, Ferrin TE (1982). A geometric approach to macromolecule-ligand interactions. Journal of Molecular Biology.

[ref-11] Kuo F, Lin W, Chen Y (2016). Affinity capture using peptide-functionalized magnetic nanoparticles to target Staphylococcus aureus. Nanoscale.

[ref-12] Liu F, Wang T, Dong X, Sun Y (2007). Rational design of affinity peptide ligand by flexible docking simulation. Journal of Chromatography A.

[ref-13] Liu R, Li X, Lam KS (2017). Combinatorial chemistry in drug discovery. Current Opinion in Chemical Biology.

[ref-14] Liu Z, Guo F, Wang F, Li TC, Jiang W (2016). 2.9 angstrom resolution Cryo-EM 3D reconstruction of close-packed virus particles. Structure.

[ref-15] Lowe CR, Burton SJ, Burton NP, Alderton WK, Pitts JM, Thomas JA (1992). Designer dyes: ‘biomimetic’ ligands for the purification of pharmaceutical proteins by affinity chromatography. Trends in Biotechnology.

[ref-16] Lund LN, Gustavsson PE, Michanel R, Lindgren J, Norskov-Lauritsen L, Lund M, Houen G, Staby A, Hilaire PM (2012). Novel peptide ligand with high binding capacity for antibody purification. Journal of Chromatography A.

[ref-17] Muegge I, Martin YC (1999). A general and fast scoring function for protein-ligand interactions: a simplified potential approach. Journal of Medicinal Chemistry.

[ref-18] Ouyang X, Handoko SD, Kwoh CK (2011). CScore:a simplle yet effective scoring function for protein—ligand binding affinity prediction using modified cmac learning architecture. Journal of Bioinformatics and Computational Biology.

[ref-19] Rarey M, Kramer B, Lengauer T, Klebe G (1996). A fast flexible docking method using an incremental construction algorithm. Journal of Molecular Biology.

[ref-20] Roberts RW, Szostak JW (1997). RNA-peptide fusions for the in vitro selection of peptides and, proteins. Proceedings of the National Academy of Sciences of the United States of America.

[ref-21] Sammond DW, Bosch DE, Butterfoss GL, Purbeck C, Machius M, Siderovski DP, Kuhlman B (2011). Computational design of the sequence and structure of a protein-binding peptide. Journal of the American Chemical Society.

[ref-22] Segalés J (2015). Best practice and future challenges for vaccination against porcine circovirus type 2. Expert Review of Vaccines.

[ref-23] Serpa G, Augusto EF, Tamashiro WM, Ribeiro MB, Miranda EA, Bueno SM (2005). Evaluation of immobilized metal membrane affinity chromatography for purification of an immunoglobulin G1 monoclonal antibody. Journal of Chromatography.

[ref-24] Smith GP (1985). Filamentous fusion phage: novel expression vectors that display cloned antigens on the virion surface. Science.

[ref-25] Thirumal Kumar D, Lavanya P, George PDC, Tayubi IA, Naveen Kumar DR, Francis Yesurajan I, Siva R, Balaji V (2017). A molecular docking and dynamics approach to screen potent inhibitors against fosfomycin resistant enzyme in clinical klebsiella pneumoniae. Journal of Cellular Biochemistry.

[ref-26] Tinberg CE, Khare SD, Dou J, Doyle L, Nelson JW, Schena A, Jankowski W, Kalodimos CG, Johnsson K, Stoddard BL, Baker D (2013). Computational design of ligand binding proteins with high affinity and selectivity. Nature.

[ref-27] Wang J, Peng X, Liu D, Bao Y, An L (2005). Synthesis of new ‘biomimetic’ dye-ligands and their application in the purification of alkaline phosphatase. Separation and Purification Technology.

[ref-28] Wang R, Lin D, Chu W, Zhang Q, Yao S (2016a). New tetrapeptide ligands designed for antibody purification with biomimetic chromatography: molecular simulation and experimental validation. Biochemical Engineering Journal.

[ref-29] Wang R, Lin D, Chu W, Zhang Q, Yao S (2016b). New tetrapeptide ligands designed for antibody purification with biomimetic chromatography: molecular simulation and experimental validation. Biochemical Engineering Journal.

[ref-30] Wang R, Lin D, Tong H, Yao S (2014). Molecular insights into the binding selectivity of a synthetic ligand DAAG to Fc fragment of IgG. Journal of Molecular Recognition.

[ref-31] Welch W, Ruppert J, Jain AN (1996). Hammerhead: fast, fully automated docking of flexible ligands to protein binding sites. Chemistry & Biology.

[ref-32] Yang S, Ouyang H, Su X, Gao H, Kong W, Wang M, Shu Q, Fu Z (2016). Dual-recognition detection of Staphylococcus aureus using vancomycin-functionalized magnetic beads as concentration carriers. Biosensors and Bioelectronics.

[ref-33] Yu Q, Wang F, Hu X, Xing G, Deng R, Guo J, Cheng A, Wang J, Hao J, Zhao H, Teng M, Zhang G (2018). Comparison of two docking methods for peptide-protein interactions. Journal the Science of Food and Agriculture.

[ref-34] Yuriev E, Ramsl PA (2013). Latest developments in molecular docking: 2010–2011 in review. Journal of Molecular Recognition.

[ref-35] Zaveckas M, Snipaitis S, Pesliakas H, Nainys J, Gedvilaite A (2015). Purification of recombinant virus-like particles of porcine circovirus type 2 capsid protein using ion-exchange monolith chromatography. Journal of Chromatography B.

[ref-36] Zhang H, Wang X, Li X, Ma Z, Feng R (2018). Construction, expression, and characterization of a single-chain variable fragment (ScFv) antibody targeting to the encephalomyocarditis virus. Journal of Medical Virology.

[ref-37] Zhao W, Shi Q, Sun Y (2014). FYWHCLDE-based affinity chromatography of IgG: effect of ligand density and purifications of human IgG and monoclonal antibody. Journal of Chromatography A.

[ref-38] Zhao Y (2018). Molecular identification and evolution analysis of f porcine circovirus type 2 isolated from Qinghai province. China Animal Health Inspection.

[ref-39] Zheng G, Lu Q, Wang F, Jin Q, Teng M, Zhang N, Ren T, Ding P, Zhang G (2017). Selection of affinity peptides for the purification potential of porcine circovirus type 2 (PCV2) Cap virus-like particles (VLPs). RSC Advances.

