# Supplementary material for: Design and preliminary application of affinity peptide based on the structure of the porcine circovirus type II Capsid (PCV2 Cap)
Source: PeerJ. 2019 Dec 5;7:e8132. doi: 10.7717/peerj.8132 (PMC6899342; doi:10.7717/peerj.8132)
Supplement: Supplemental Information 1 [file peerj-07-8132-s001.doc]

1. **The preparation of PCV2 Cap protein biochip**

The purified PCV2 Cap protein was covalently immobilized on a carboxyl gold nanoparticles (COOH-AuNPs) chip. All the steps were performed according to manufacturer’s instructions. First, the activation of carboxyl chip: the chip is activated by the EDC/NHS reagent in the amine coupling kit. Second, the coupling of protein: the PCV2 Cap protein (50 μg/mL) was covalently attached to COOH-AuNPs chip. The last step is to block the chip with blocking buffer. In every step, The 300 μL sample was flowed through the chip for 5 min at a constant flow rate of 20 μL/min. The successful immobilization of the PCV2 Cap was confirmed by the observation of a ~500 pm singal increase in the sensor chip.

**Figure. 1** **The** **preparation of PCV2 Cap protein biochip**

**Fig. 1**


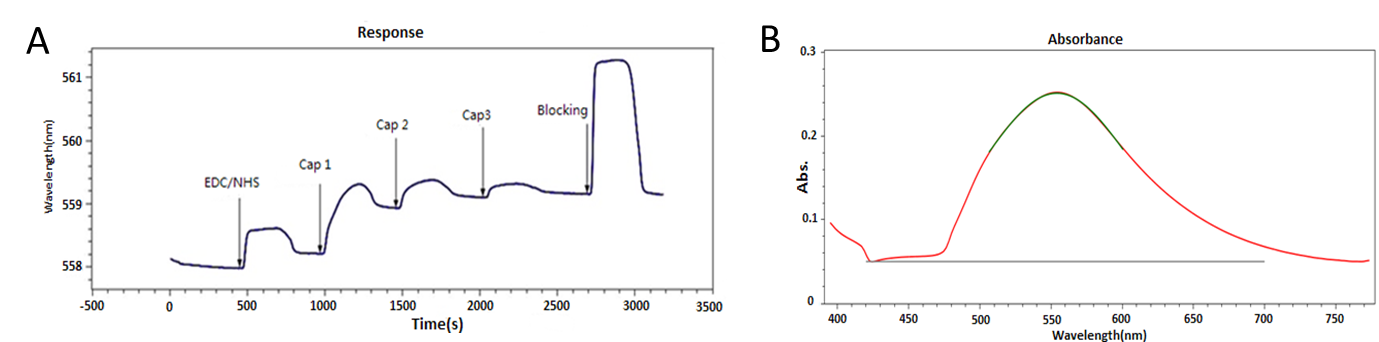


**Figure. 1** The results of the PCV2 Cap protein coupled to the COOH-chip and the resonance absorption peak of the PCV2 Cap protein.

Fig. 1 A showed that the PCV2 Cap was successfully immobilized by a wavelength value greater than 500 pm. Fig. 1B: The maximum resonance absorption peak of PCV2 Cap protein on LSPR, is around 550 nm.

1. **The size of NaMB and the coupling process of NaMB-L11 affinity adsorbent**

The size of NHS agarose magnetic beads (NaMB) is 25 μm and its surface contains NHS groups. The coupling process of the affinity adsorbent NaMB-L11 was as follows：(according to the instructions of the NaMB kit, lightly modified).

(1) The 100 μL of NaMB was taken into a 1.5 mL EP tube, washed twice with 500 μL of absolute ethanol, and the supernatant was removed by magnetic separation.

(2) The amount of pre-designed peptide L11 was added (the peptide L11 dissolved in the coupling buffer), mixed and rotated at room temperature for 2 h. The supernatant was removed by magnetic separation.

(3) Subsequently, the 500 μL of blocking buffer was added, mixed and rotated for 2 h at room temperature. The supernatant was removed by magnetic separation.

(4) Finally, the 500 μL of washing buffer was added and washed the magnetic beads for about 30 s (repeated 3~5 times)**.** The supernatant was removed by magnetic separation.

1. **The** **expression of recombinant PCV2 Cap protein**

First, the genomic DNA of the PCV2 Cap protein was cloned in pET28a vector (pET28a-Cap). Second, the plasmid pET28a-Cap was transformed into the host cell BL21. Finally, the Isopropyl-beta-D-thiogalactopyranoside (IPTG, 1 mM) was added to induce the expression, which reacted for 12 h 28°C. Meanwhile, the pET28 empty vector was used as a control.

**Figure. 2 SDS-PAGE analysis of the PCV2 Cap protein expressed in *E. coli* BL21**.

**Fig. 2**


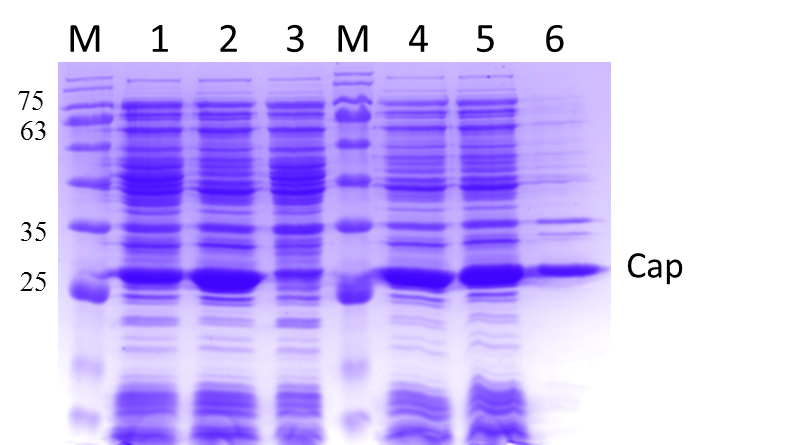


**Fig.** **2** **SDS-PAGE analysis of the PCV2 Cap protein expressed in *E. coli* BL21**.

Lane 1: pET28a-Cap cells before induction; Lane 2: pET28a-Cap cells after induction; Lane 3: pET28a vector control; Lane 4: the total cells of pET28a-Cap after ultrasonication; Lane 5: the supernatant of pET28a-Cap after ultrasonication; Lane 6: the precipitation of pET28a-Cap after ultrasonication.

Fig. 2A showed that the PCV2 Cap protein was successfully expressed in BL21 in soluble form. Its apparent molecular weight is about 26 kDa, which is in accordance with the expected size. The solubility and insolubility of pET28a-Cap protein were analyzed in SDS-PAGE by centrifugation for 12,000 rpm×20 min. The PCV2 Cap contained in the supernatant is soluble, and the PCV2 Cap in the precipitate is insoluble.

1. **The calculation of ligand density (Indirect measurement of ligand densities)**

The ligand density is obtained by the formula:


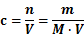


Where C represents the mass concentration of the solution; n represents the amount of matter; V represents the volume of the solution; m represents the mass; M represents the relative molecular weight of the solute（the relative molecular mass of the L11 peptide is 1199）. The calculated ligand densities at different coupling ratios are shown in Table 1.

**Table. 1 The ligand density at different coupling ratios.**

| Coupling ratio | Peptide density |
| --- | --- |
| 600 µg/100µL | 5 mmol/L |
| 300 µg/100µL | 2.5 mmol/L |
| 150 µg/100µL | 1.25 mmol/L |
| 75 µg/100µL | 0.63 mmol/L |
| 37.5 µg/100µL | 0.32 mmol/L |
| 18.8 µg/100µL | 0.16 mmol/L |
| 9.4 µg/100µL | - 1. mol/L |

1. **The affinity of 67 peptide binding with the PCV2 Cap was analyzed via ELISA.**

The affinity of 67 peptide sequences to PCV2 Cap was qualitatively determined by ELISA; thirteen peptides (named L1-L13) with the highest OD value (OD﹥1.0) were selected as candidate peptides (see Fig. 3 ). The sequence numbers of the following peptides are 8, 12, 16, 23, 24, 27, 30, 32, 37, 45, 54, 58 and 61, which are named L1-L13 in turn .

**Fig .3**


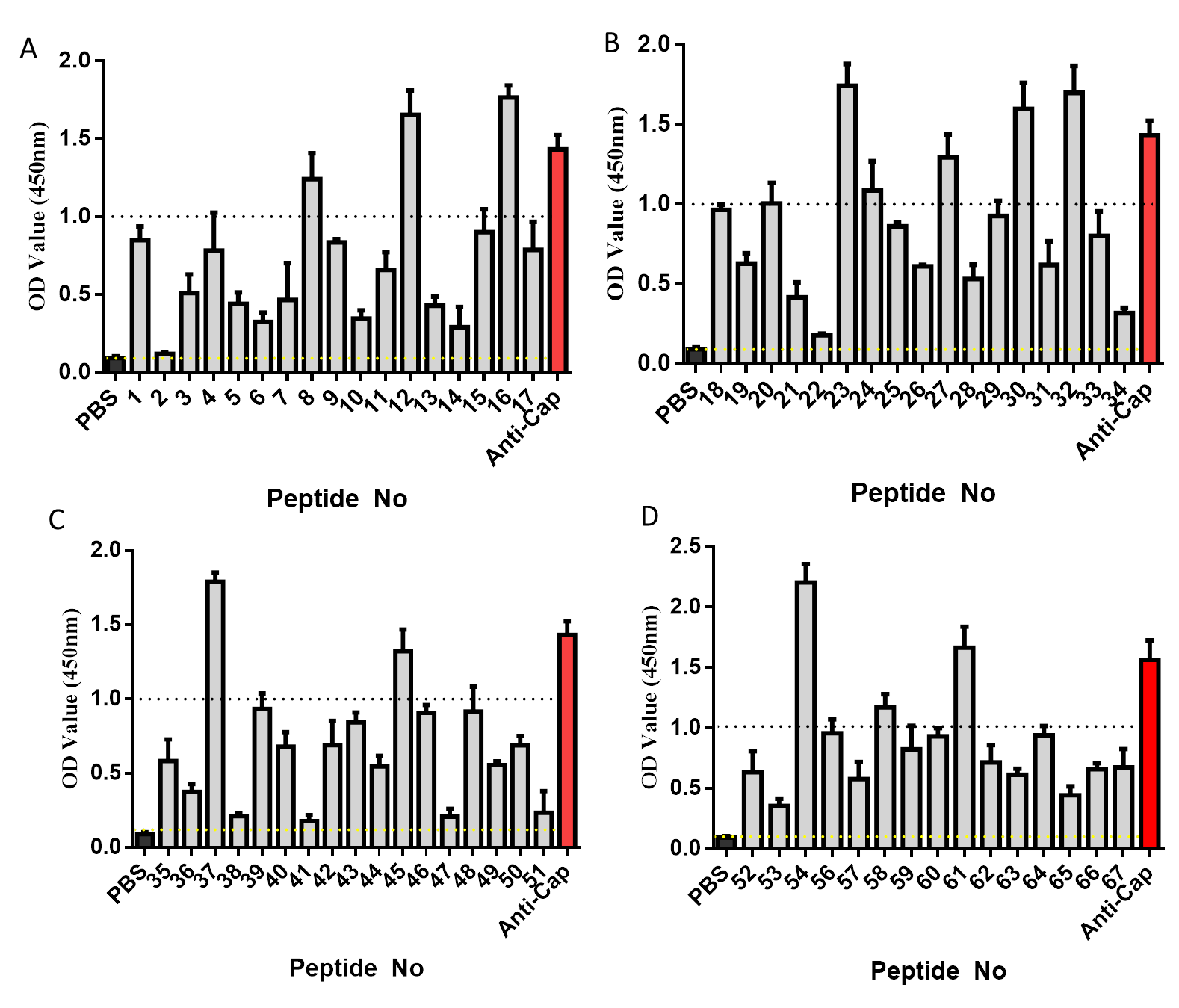


**Figure. 3** The affinity of 67 peptide binding with the PCV2 Cap was analyzed via ELISA. (A), (B), (C) and (D) sequentially showed the OD values of 67 peptide sequences.

1. **1:1 binding interaction model**

Dissociation constant Kd represents the ratio of AB (ie, AB) dissociated in unit time to the initial AB before dissociation, which represents the speed of the dissociation reaction.Equilibrium dissociation constant K_D_ =k_d_/k_a_, K_D_ unit is M (mol/L)

1:1 binding interaction model represents the binding mode of ligand and target according to 1:1. The1:1 binding interaction model is a differential equation:

dY/dt = (k a * c - k d )* Y

Y(t=0) = 0 , signal from saturated target = Bmax

Y is the recorded signal and c is the concentration of ligand in the bulk liquid.
