# Supplementary material for: Design and preliminary application of affinity peptide based on the structure of the porcine circovirus type II Capsid (PCV2 Cap)
Source: PeerJ. 2019 Dec 5;7:e8132. doi: 10.7717/peerj.8132 (PMC6899342; doi:10.7717/peerj.8132)
Supplement: Supplemental Information 2 [file peerj-07-8132-s002.doc]

**Data 1. The OD values of 13 affinity peptides interacting with PCV2 Cap protein**

| SampleNo | OD 1 | OD 2 | OD 3 |
| --- | --- | --- | --- |
| PBS | 0.132 | 0.092 | 0.102 |
| L1 | 1.621 | 1.337 | 1.278 |
| L2 | 1.839 | 1.678 | 1.732 |
| L3 | 1.028 | 1.312 | 0.981 |
| L4 | 2.19 | 1.939 | 1.893 |
| L5 | 0.931 | 0.821 | 0.756 |
| L6 | 1.412 | 1.237 | 1.264 |
| L7 | 1.731 | 1.869 | 1.746 |
| L8 | 1.321 | 1.654 | 1.321 |
| L9 | 2.298 | 2.16 | 2.267 |
| L10 | 1.513 | 1.736 | 1.567 |
| L11 | 2.613 | 2.454 | 2.535 |
| L12 | 1.093 | 1.123 | 1.067 |
| L13 | 1.934 | 2.0631 | 1.875 |

**Data 2. The P/N of affinity peptides**

| Peptide NO | Average P | Average N | P/N |
| --- | --- | --- | --- |
| L4 | 1.537 | 0.31 | 4.958065 |
| L7 | 1.294 | 0.284 | 4.556338 |
| L9 | 1.462 | 0.318 | 4.597484 |
| L11 | 1.755 | 0.315 | 5.571429 |
| L13 | 1.283 | 0.362 | 3.544199 |

**Data3. The cross-reactivity of affinity peptides**

| Sample No | PEDV-S | | PRRS-GP5 | | BSA | |
| --- | --- | --- | --- | --- | --- | --- |
| L4 | 0.7246 | 0.635 | 0.884 | 0.736 | 0.513 | 0.437 |
| L7 | 0.823 | 0.765 | 0.578 | 0.456 | 0.382 | 0.419 |
| L9 | 0.437 | 0.476 | 0.237 | 0.0.296 | 0. 348 | 0.279 |
| L11 | 0.573 | 0.678 | 0.872 | 0.7827 | 0.297 | 0.476 |
| L13 | 0.898 | 0.946 | 0.645 | 0.754 | 0.367 | 0.468 |

All analyses were conducted using GraphPad Prism version 5.0 software and Excel. Values were expressed as mean ± SEM. Cross- reactivity between groups was performed with two-way ANOVA. Statistical significance was determined at *P*＜0.05 (*), *P*＜0.01 (**), or *P*＜0.001 (***).

**Data. 4 Kinetic constants for LSPR analysis**

L4- LSPR data.

| Curve name | ka (1/(M*s)) | kd (1/s) | KD (M) |
| --- | --- | --- | --- |
| L4-1.56 µM fitted | 1.92E+03 | 2.36E-03 | 1.23E-06 |
| L4-3.125 µM fitted | 1.92E+03 | 2.36E-03 | 1.23E-06 |
| L4-6.25 µM fitted | 1.92E+03 | 2.36E-03 | 1.23E-06 |
| L4-12.5 µM fitted | 1.92E+03 | 2.36E-03 | 1.23E-06 |

Data were fit to a 1:1 interaction model using the analysis software TraceDrawer.

L7 -LSPR data

| Curve name | ka (1/(M*s)) | kd (1/s) | KD (M) |
| --- | --- | --- | --- |
| L7-2 µM fitted | 8.56E+02 | 9.10E-04 | 1.06E-06 |
| L7-16 µM fitted | 8.56E+02 | 9.10E-04 | 1.06E-06 |
| L7-32 µM fitted | 8.56E+02 | 9.10E-04 | 1.06E-06 |
| L7-8 µM fitted | 8.56E+02 | 9.10E-04 | 1.06E-06 |

Data were fit to a 1:1 interaction model using the analysis software TraceDrawer.

L9- LSPR data

| Curve name | ka (1/(M*s)) | kd (1/s) | KD (M) |
| --- | --- | --- | --- |
| L9-25 µM fitted | 8.55E+02 | 5.74E-03 | 6.71E-06 |
| L9-0.78 µM fitted | 8.55E+02 | 5.74E-03 | 6.71E-06 |
| L9-12.5 µM fitted | 8.55E+02 | 5.74E-03 | 6.71E-06 |

Data were fit to a 1:1 interaction model using the analysis software TraceDrawer.

L11- LSPR data

| Curve name | ka (1/(M*s)) | kd (1/s) | KD (M) |
| --- | --- | --- | --- |
| L11-3.125µMfitted | 1.01E+04 | 1.04E-03 | 1.03E-07 |
| L11-6.25 µM fitted | 1.01E+04 | 1.04E-03 | 1.03E-07 |
| L11-12.5 µM fitted | 1.01E+04 | 1.04E-03 | 1.03E-07 |

Data were fit to a 1:1 interaction model using the analysis software TraceDrawer.

L13- LSPR data

| Curve name | ka (1/(M*s)) | kd (1/s) | KD (M) |
| --- | --- | --- | --- |
| L13-12.5 µM fitted | 3.35E+02 | 3.34E-03 | 9.97E-06 |
| L13-25 µM fitted | 3.35E+02 | 3.34E-03 | 9.97E-06 |
| L13-50 µM fitted | 3.35E+02 | 3.34E-03 | 9.97E-06 |

Data were fit to a 1:1 interaction model using the analysis software TraceDrawer.
